# New Insights into Non-Dietary Treatment in Celiac Disease: Emerging Therapeutic Options

**DOI:** 10.3390/nu13072146

**Published:** 2021-06-23

**Authors:** Verónica Segura, Ángela Ruiz-Carnicer, Carolina Sousa, María de Lourdes Moreno

**Affiliations:** Departamento de Microbiología y Parasitología, Facultad de Farmacia, Universidad de Sevilla, 41012 Sevilla, Spain; vsegura@us.es (V.S.); acarnicer@us.es (Á.R.-C.); csoumar@us.es (C.S.)

**Keywords:** celiac disease, gluten-free diet, gluten, gliadin, gluten immunogenic peptides, non-dietary therapies

## Abstract

To date, the only treatment for celiac disease (CD) consists of a strict lifelong gluten-free diet (GFD), which has numerous limitations in patients with CD. For this reason, dietary transgressions are frequent, implying intestinal damage and possible long-term complications. There is an unquestionable need for non-dietary alternatives to avoid damage by involuntary contamination or voluntary dietary transgressions. In recent years, different therapies and treatments for CD have been developed and studied based on the degradation of gluten in the intestinal lumen, regulation of the immune response, modulation of intestinal permeability, and induction of immunological tolerance. In this review, therapeutic lines for CD are evaluated with special emphasis on phase III and II clinical trials, some of which have promising results.

## 1. Introduction

Celiac disease (CD) is a chronic immune-mediated enteropathy triggered by exposure to dietary gluten in genetically predisposed individuals [1,2]. The pooled global prevalence of CD has been reported to be approximately 1%, however, the prevalence values for CD varies in South America, Africa, North America, Asia, Europe, and Oceania; the prevalence is higher in female vs. male individuals and is 4–8 times higher among non-Hispanic white people compared with other races. Moreover, there has been an increase in the diagnosis rate in the last 10 years [3,4,5,6,7]. CD is characterized by intestinal and/or extraintestinal manifestations, elevation of specific antibodies such as anti-gliadin and anti-tissue transglutaminase (anti-tTG), and the presence of HLA-DQ2/DQ8 haplotypes [8,9,10,11].

Gluten is a complex mixture of seed storage proteins known as prolamins, found in cereals grains such as wheat, barley, rye, oats, and their derivatives. The viscoelastic network generated by gluten enables an excellent aerated structure, contributing to the baking quality of these cereals [3,4,5,6,7,8,9,10,11,12]. Gluten proteins are characterized by high proline and glutamine content. Therefore, these proteins are partially degraded to peptides by digestive proteases of the gastrointestinal track that persist in the intestine and potentiate their deamidation through tTG [13]. 

The prevailing hypothesis of immunopathogenesis is the two-signal model, which establishes that gluten has a dual effect on the duodenum of celiac patients mediated by innate and adaptive immune systems [14,15]. Certain peptides, such as the 19-mer gliadin peptide, trigger an innate immune response mainly characterized by the production of interleukin-15 (IL-15) by epithelial cells and the disruption of the epithelial barrier caused by increased permeability and induction of enterocyte apoptosis [16,17]. Consequently, other peptides such as the 33-mer gliadin can now reach the lamina propria to be deamidated by tTG, providing a negative charge to gliadin peptides that activate the immune-adaptive system. The affinity of the HLA-DQ2/8 peptide is enhanced and expressed on the surface of dendritic cells (DCs) [18,19,20]. DCs present a gluten antigen to T-cells and drive the progression of the proinflammatory response, thereby contributing to the symptomatology of the disease [21,22].

## 2. Gluten-Free Diet: Challenge and Gluten Exposure

Currently, the only available treatment for CD is a strict, lifelong gluten-free diet (GFD). Dietary gluten restriction is a safe and effective therapy; however, unintentional gluten exposure on a GFD is common and intermittent. Recent findings suggest that most CD patients can only attain a gluten-reduced diet instead of the recommended strict GFD. Gluten exposure may be more common than realized and is distinct from lapses in an otherwise intentionally strict GFD [23,24]. 

Among the main causes of gluten exposure in a GFD is the ubiquitous nature of gluten, food cross-contamination, and the limitations and socio-emotional toll [25]. In addition, many of the manufactured gluten-free products tend to be less healthy than their gluten analogues since high amounts of lipids, sugars, and other additives are incorporated in their production to simulate the viscoelastic properties of gluten proteins [26]. Although it is well known that legislation on the labeling of gluten-free products is based on the limitation of 20 parts per million (ppm) of gluten [27], there is no clear consensus on the safe amount of daily gluten intake due to the threshold for triggering symptoms has interindividual variability. Total daily gluten consumption that seems to be safe for most CD patients is <50 mg gluten; nevertheless, little amounts as 10 mg of daily gluten for some CD patients could promote development of intestinal mucosal abnormalities [28].

**Figure 1:**
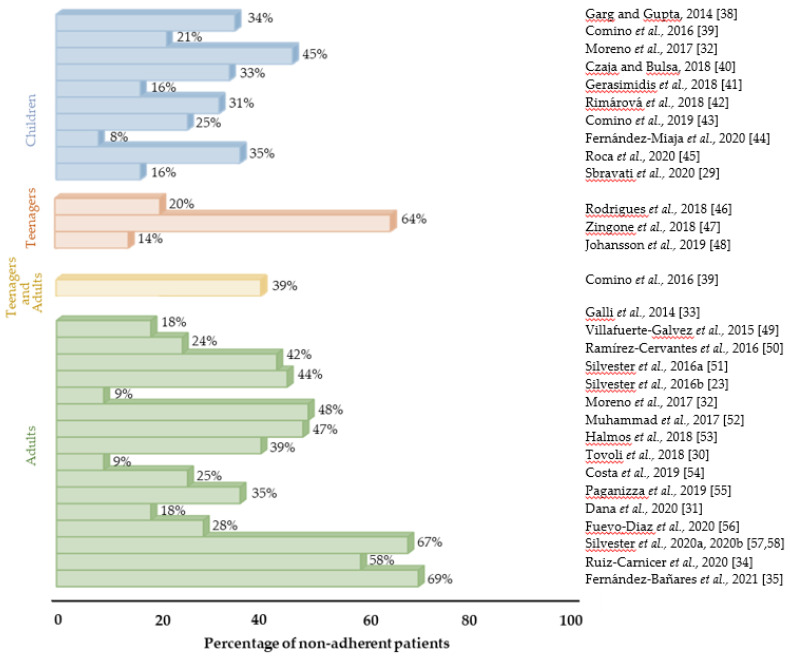
Studies reporting gluten exposure rates in CD patients on a supposed GFD. CD, celiac disease; GFD, gluten-free diet [23,29,30,31,32,33,34,35,36,37,38,39,40,41,42,43,44,45,46,47,48,49,50,51,52,53,54,55,56,57,58].

Several studies based on nutritional questionnaires, serological tests, and evaluating gluten immunogenic peptides in feces and urine, have reported variable gluten exposure rates in patients with CD, reaching up to 69% in adults, 64% in adolescents, and 45% in children (Figure 1) despite their best efforts to avoid it. Studies reporting gluten exposure rates may compromise high rates of ongoing symptoms [29,30,31] and enteropathy [32,33,34,35] in patients with CD, leading to comorbidities such as anemia, severe malabsorption, and various forms of malignancies [36]. Hence, it is important to drive efforts to develop non-dietary adjunctive or alternative therapies for CD treatment [37]. Recently, researchers have attempted to meet the requests of celiac patients seeking therapies aside from GFD. In this review, we summarize the spectrum of potential therapeutic agents to improve CD management and their research status, highlighting several drug candidates in phase II/III clinical trials. 

## 3. Potential Alternative or Adjuvant Non-Dietary Treatments for CD

**Figure 2:**
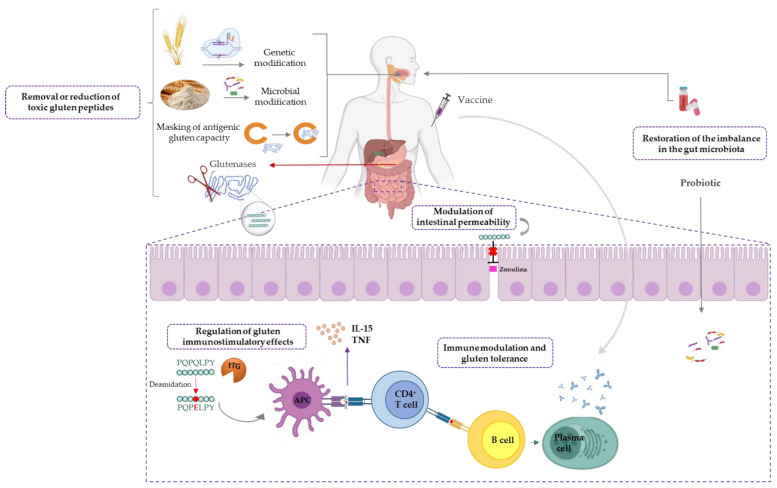
Emerging therapeutic approaches for non-dietary CD treatment. APC, antigen-presenting cell; CD, celiac disease; IL-15, interleukin 15; TNF, tumor necrosis factor; tTG, tissue transglutaminase.

The emerging therapeutic options for CD can be broadly classified into one of the following strategies—(1) removal of toxic gluten peptides before reaching the intestine, (2) regulation of the immunostimulatory effects of toxic gluten peptides, (3) modulation of intestinal permeability, (4) immune modulation and induction of gluten tolerance, and (5) restoration of the imbalance in the gut microbiota (Figure 2). 

Many of the sequential steps in CD pathogenesis are well-elucidated; hence, multiple well-defined targets for research and drug development are available (Table 1). Likewise, therapies focused on the regulation of the immunostimulatory effects have been described for other related pathologies, and due to their efficacy, their indications have been extended to CD. 

### 3.1. Removal or Reduction of Toxic Gluten Peptides

Therapies aimed at eliminating or reducing gluten peptides can act in food before marketing, during digestion in the human tract, or masking the antigenic capacity before reaching the intestinal mucosa. 

#### 3.1.1. Genetic Modification of Gluten-Containing Cereals

The development of cereals with reduced or absent immunogenic gluten proteins is important for the management of CD. The wheat variants currently used have been reported to be more immunogenic than the ancestral or wild variants such as those belonging to the genera *Tritordeum* or *Triticum* [92,93]. Genetic advances in plants have successfully allowed the production of wheat lines with very low or completely lacking gluten content through the hybridization of wheat species [94]. A recent study described the traditional breeding and characterization of a novel ultralow gluten barley variety in which the gluten content was reduced to below 5 ppm by combining three recessive alleles, which act independently to lower the hordein content in the parental varieties [59].

RNA interference to silence the expression of gluten proteins that contain immunogenic epitopes for CD has been employed as a genetic engineering strategy [95]. This approach has allowed the development of wheat lines that contain very few immunogenic epitopes of CD, and, therefore, could be consumed by patients with non-celiac gluten sensitivity, since it produces no adverse clinical symptoms [96,97]. Currently, several studies are in progress to understand the effects of these new lines in patients with CD. 

The use of CRISPR/Cas9 (Clustered Regulatory Interspaced Palindromic Repeats associated protein 9) technology can precisely and efficiently reduce the amount of α-gliadin in the seed kernel, providing bread and durum lines with reduced immunoreactivity for the celiac community [60,94]. However, it is likely that the deleted gliadin genes need to be replaced by non-immunogenic gliadin variants to obtain adequate elasticity. Additionally, governmental regulations for genetic modification of food products require expensive and time-consuming food safety assessments to be met before product marketing [94].

#### 3.1.2. Microbial Gluten Modification

The addition of diverse microorganisms in sourdough for fermentation has been studied because it contains proteases capable of hydrolyzing gluten peptides rich in glutamine and proline residues. Diverse studies using species of the genus *Lactobacillus* have reported that this baking method could obtain safe breads for celiac patients [62,98]. The well-known probiotic preparation VSL#3 comprises eight strains belonging to the genera *Bifidobacterium, Lactobacillus,* and *Streptococcus*. This cocktail was assayed during the food processing step and produced tolerable predigested gliadins without α-gliadin peptides p62-75 and 33-mer, but with the palatability of gluten-free products [99]. This study demonstrated the improvement in the symptoms of adult CD patients with irritable bowel syndrome (IBS) [100]. Furthermore, the probiotic preparation was capable of stabilizing intraepithelial junctions, promoting the barrier effect that prevents the entry of toxic peptides into the lamina propria [91,101]. However, individual probiotic strains are inadequate to break down gliadin compared to the group efficacy [101,102]. 

Another investigated approach in the preclinical phase consists of the pretreatment of flours or sourdoughs with microbial transglutaminase (m-TG) and N-methyl-lysine [103,104]. The use of N-methyl lysine and m-TG derived from *Streptomyces mobaraensis* provoked gluten modification and loss of affinity for the HLA-DQ2 molecule, which leads to less activation of intestinal T lymphocytes [105]. Although the effect of standard bakery concentrations of microbial transglutaminase (m-TG) in wheat bread preparation on the immunoreactivity of sera of CD patients was investigated, its use in food preparation remains a subject of debate [63].

#### 3.1.3. Masking of Antigenic Gluten Capacity 

The gluten-binding polymer BL-7010 or copolymer poly-hydroxyethylmethacrylate-co-styrene sulfonate (P-HEMA-co-SS) complex is a non-absorbable synthetic origin blocking agent that binds intraluminal gluten [64]. Therefore, digestive enzymes cannot access the cleavage sites, preventing the degradation of immunogenic peptides that are not absorbed by the intestine and do not induce an immune response. The effect of BL-7010 has been investigated in intestinal biopsy samples from patients with CD [64,65,106]. Attenuation of the immune response and the high safety profile in animal models were observed; however, this phase II therapy was discontinued in 2017. 

Recent studies have developed neutralizing anti-gliadin antibodies extracted from egg yolk (AGY-010). IgY antibodies have shown effectiveness in neutralizing and absorbing gliadin, as well as resistance to stomach conditions [66]. This therapy is currently in phase II studies and a study is ongoing to evaluate its efficacy and safety in CD patients [107]. As the use of egg yolk antibodies might be inefficient for large-scale clinical production, parallel recombinant antibody fragments in single-chain format have been produced for the same purpose [108].

#### 3.1.4. Luminal Gluten Detoxification 

Oral enzyme therapy is focused on the inactivation of gluten peptides in the human gastrointestinal tract before reaching the intestine. Gluten-degrading enzymes seem to hold the most promise as attractive therapies for helping patients with CD to avoid accidental gluten ingestion and to promote better overall health. A prerequisite is that such enzymes should be active under gastro-duodenal conditions, quickly neutralize the T-cell-activating gluten peptides and be safe for human consumption [67,68,70,109].

Glutenases have been identified in bacteria, fungi, plants, and even insects (Table 2). Although the enzymes studied are endopeptidases, interesting exopeptidases have also been described [110]. Endopeptidases are further subdivided depending on their catalytic mechanism; among them, prolyl endopeptidases (PEPs) are especially effective in hydrolyzing peptide bonds on the carboxyl side of internal proline residues in gluten-derived oligopeptides [69]. The potential synergism between gluten-degrading enzymes that differ in their cleavage specificities and optimum pH values raises the possibility of a mixture that would more effectively eliminate the antigenicity of ingested gluten fractions [111]. 

Among the bacterial enzymes capable of degrading gluten, PEPs are produced by *F. meningosepticum* [68,69], *S. capsulata* [70,71] and *M. xanthus* [69]. These three enzymes showed high specificity against reference chromogenic substrates and the potential to successfully degrade the immunogenic sequences of gluten. The cysteine endoprotease EP-B2 and PEP from *F. meningosepticum* complement each other in terms of their gluten hydrolytic properties; however, significant efforts have been made to increase their thermostability to be suitable for industrial applications [111]. 

Fungal PEP from *A. niger*, known as AN-PEP, exhibits post-proline cleavage activity and is highly efficient in degrading gluten [72]. A clinical study with Tolerase G, an AN-PEP-based supplement, reduced the amount of gluten exposed in the duodenum efficiently, despite not completely degrading the gluten [72]. The enzyme preparation consisting of AN-PEP from *A. niger* and DPP-IV from *A. oryzae* (STAN 1) administered orally in celiac patients appeared to be modest because of the non-specificity of AN-PEP and the very limited proteolytic effect of DPP-IV. Therefore, these studies were stalled in phase II in 2017. In the genus *Aspergillus,* another enzyme was detected with gluten-degrading activity, termed aspergillopepsin (ASP) from *A. niger,* although ASP needs to be used as a complementary enzyme because of its incomplete degradation [118]. In this sense, a dietary supplement has been widely used in the food and feed industry containing ASP from *A. niger* and DPP-IV from *A. oryzae*, which successfully degraded small amounts of gluten in vitro [119]. 

As previously argued, the combination of enzymes appears to be a future direction in enzyme therapy. The enzymatic cocktail, latiglutenase or IMGX-003 (formerly ALV003), consists of a 1:1 combination of cysteine endoprotease from barley EP-B2 (IMGX-001), and PEP from *S. capsulate* SC-PEP (IMGX-002). A phase II gluten challenge to investigate its effect on both mucosal and symptomatic protection in CD patients is in progress. Initial findings with latiglutenase have been shown to mitigate gluten-induced intestinal mucosal injury as well as to reduce the severity and frequency of symptoms in patients with CD [73,125]. Evidence of symptom relief was particularly pronounced in patients with positive serology despite following a GFD [61,126,127]. 

An engineered synthetic gluten-degrading enzyme, KumaMax, with technological improvements, is being studied. KumaMax showed similar in vitro results to IMGX-003, although it is still under development [128]. The gluten-degrading enzyme subtilisin-A (Sub-A) from *B. licheniformis* was modified by PEGylation and subjected to microencapsulation. The effectiveness was confirmed in vitro and in vivo and showed a significant increase in protection against acid exposure [113]. 

Investigating the effect of glutenases on the symptoms and biomarkers in CD patients with randomized, placebo-controlled studies is mandatory; however, this is not as straightforward as it might seem.

### 3.2. Immune Response Regulation

As inflammatory mediators are common in CD and other gastrointestinal pathologies, certain therapies aimed at avoiding chronic gastrointestinal inflammation could be applied in CD. 

tTG plays a critical role in the pathogenesis of CD through the deamidation and transamidation of gluten peptides, which leads to an immune response with inflammation of the intestinal mucosa [129,130]. Hence, the inhibition of tTG results in the abolishment of gluten peptide presentation by HLA-DQ2/DQ8, preventing the immune response. Three varieties of tTG-2 inhibitors have been well described, namely, irreversible inhibitors, reversible inhibitors, and competitive amine inhibitors. ZED-1227 is a highly specific orally active irreversible inhibitor with promising preliminary preclinical results. A phase II clinical study with ZED-1227 is ongoing in EU countries in healthy volunteers [76]. Nevertheless, tTG plays a critical role in gut wound healing, and its safety and efficacy require further study [131]. Among competitive inhibitors, cystamine is currently the only competitive commercially available tTG-2 inhibitor despite that it has not been explored for its potential role in CD. Recently, Palansky et al. [132] discovered that disulfiram, an FDA-approved drug for alcohol abuse, is also a tTG inhibitor. This is the first clinically approved compound to show human tTG inhibitory activity, raising further interesting possibilities for the future in terms of tTG inhibition as a therapeutic strategy in CD [133].

Another attractive therapeutic target to prevent the activation of the immune response is the HLA-DQ2 blocker. Gluten-like molecules in which proline residues have been replaced by azidoprolines do not elicit an immune response in T-cells isolated from individuals with CD [8]. Cyclic and dimeric peptides have also been developed that bind DQ2, partially blocking T-cell proliferation and antigen presentation. However, these molecules do not fully block the activation of T-cells; therefore, other nontoxic antagonists with high affinity are currently being studied [129].

Some studies have highlighted the role of IL-15 and the receptor activator NKG2D and other immune soluble factors as targets of CD treatment. IL-15 plays a critical role in the activation of intraepithelial lymphocytes and participates in both innate and adaptive responses. NKG2D is the receptor of T-cells and natural killer cells [134]. The first monoclonal antibody (moAb) studied against the IL-15 receptor was Hu-Mik-Beta-1, and positive results were obtained in refractory CD. However, this therapy was stuck in phase I. Second, PRV-015 (also known as AMG 714) is a fully human moAb that has emerged as a leading investigational candidate for nonresponsive CD (NRCD), in which patients maintain disease activity despite an ongoing GFD. Phase II studies have shown a reduction in inflammation and symptoms in a clinical trial with patients with refractory CD type 2 [80]. Lastly, CALY-002 is a moAb whose safety, tolerability, pharmacokinetics, and pharmacodynamics are being evaluated in phase II studies in both CD and eosinophilic esophagitis [135].

Tumor necrosis factor (TNF)-γ secreted by T-cells in response to gluten is another therapeutic target under study. Fontolizumab was initially developed for inflammatory bowel disease (IBD) treatment and has been proposed for CD, although clinical trials for this indication have not yet been registered. Infliximab and adalimumab moAbs targeting TNF-α have been used in clinical practice for IBD and could be useful in treating CD [76,136]. 

Among T-cell-targeted therapies aimed at blocking lymphocyte recruitment, natalizumab is an anti-α4 used in Crohn’s disease and could be useful in CD, although its side effects are very high [79,137]. Vedolizumab is scheduled to start phase II studies that block α4β7 integrin [138]. In addition, chemokine receptor inhibitors such as CXCR3 and its specific ligands CXCL10 and CXCL11 have also been studied [79]. These molecules are among the main determinants in the recruitment of immune cells to the intestinal lamina propria and are involved in the uptake of lymphocytes in the presence of gliadin peptides. CCL25 and its receptor CCR9 appear to be a therapeutic alternative in the future, although to date it has only been studied in animal models with Crohn’s disease [139,140].

Anti-inflammatory drugs such as corticosteroids and budesonide are generally used to treat the symptoms of refractory CD. Likewise, mesalazine has been proposed, although it must be remembered that most of these formulations are prepared to be released in the colon and the inflammation in CD affects the small intestine [66]. Recent studies have shown that mesalazine has a beneficial effect on the molecules and biological mediators of inflammation that occur in the mucosa of celiac patients [81].

### 3.3. Barrier Enhancing Therapies

Increased intestinal permeability has been implicated in CD due to both transcellular and paracellular epithelial permeability, with apical junctional protein complexes called tight junctions being key components in the latter process [141].

Larazotide acetate, formerly known as AT-1001 or INN-202, is a locally acting octapeptide with a sequence analogous to a portion of *Vibrio cholerae* zonula occludens toxin [141]. In cultured intestinal epithelial monolayers, larazotide acetate enhanced actin rearrangement and prevented the disassembly of tight junctions [142,143]. In addition, larazotide acetate prevents the passage of gluten peptides to the lamina propria by closing the intercellular junctions of the enterocytes, which could help prevent the development of the immune cascade in celiac patients. Therefore, larazotide acetate is the most advanced experimental drug, showing a reduction in symptoms as well as a reduction in anti-tTG antibody levels. Three phase II studies of larazotide acetate have been completed and published in CD patients undergoing a gluten challenge, but only an excellent safety profile and efficacy with low dose have been reported in patients with NRCD. Therefore, larazotide acetate has moved forward to a phase III registration study for this indication [82,83,144].

### 3.4. Immunomodulation and Gluten Tolerance

Vaccine therapy is the preferred option among alternative treatments to a GFD in patients with CD. It is based on immunization with gluten epitopes, which induces the expansion of regulatory T-cells, restoring oral tolerance to gluten [145]. The Nexvax2 vaccine (ImmusanT, Cambridge, MA, USA) comprises the use of three gluten epitopes chosen based on a study by Tye-Din et al. [145]. This study examined epitopes within wheat, barley, and rye with the ability to induce and stimulate T-cells isolated from the serum of patients with CD on a gluten-containing diet. Nexvax2 is one of several CD drugs that has reached phase II clinical trials [141]. However, although Nexvax2 showed a good safety profile, its efficacy has yet to be demonstrated. Nexvax2 is specific only for individuals with the HLA-DQ2 genotype. Therefore, another vaccine should be investigated in patients with HLA-DQ8 genotyping [84,85].

Biodegradable nanoparticles encapsulated with gliadin proteins TAK-101 (formerly known as CNP-101 and TIMP-GLIA) seem to be a first-in-class agent that induces antigen-specific immune tolerance to CD [141]. TAK-101 binds inflammatory cells to initiate tolerogenic immune reprogramming. According to the clinicaltrials.gov, the phase II developmental trial of TAK-101 for treating patients with CD was estimated to be completed in July 2019, but it is still in the active phase, not the recruiting phase [146].

A new therapy in phase I focuses on restoring normal immune tolerance by targeting specific receptors in the liver, named KAN-101 [141]. The tolerogenic nanoparticles for intravenous injection trigger a cascade of events that drive the re-education of T-cells so that they do not respond to gluten antigens [87].

The administration of *N. americanus* infective larvae in patients with CD interferes with the host immune response due to its survival in the intestine. Studies of duodenal biopsies from CD individuals infected with *N. americanus* and exposed to gluten have shown a reduction in the production of IL-2, IFN-γ, and IL-17. In addition, the absence of histological lesions and even a decrease in anti-tTG antibody levels have been demonstrated [88]. *N. americanus* is currently in phase II clinical trials, although problems with CD patient acceptance for routine clinical use are anticipated [66,147].

Finally, other studies based on the tolerance of the mucosa to genetic modification are in the initial phase of investigations. These studies specifically focused on organoids derived from the human intestine, providing a model to study the response to gluten and the effects of molecules derived from the microbiota in patients with CD [89].

### 3.5. Restoration of the Imbalance in the Gut Microbiota

The gut microbiota is involved in the initiation and perpetuation of intestinal inflammation in several chronic diseases. Indeed, several studies have identified certain microorganisms in CD patients and healthy subjects. Therefore, alteration of the microbiota could play a significant role in the pathogenesis of CD. Recent studies have focused on the role of the gut microbiota in CD and the complex relationship between its composition, genetic background, GFD, and persistence of clinical symptoms [90,148]. The specific mechanisms by which microorganisms can participate in the development of responses to gluten are broad and include the metabolism of trigger antigen responses, enhancement of the intestinal barrier, and modulation of adaptive and innate immune responses [149]. 

Recent data have shown that genetics (HLA-DQ-2 or DQ-8) may predispose individuals with CD to dysbiosis [90,148]. Palma et al. [150] studied the effects of following a GFD on the composition of gut microbiota in healthy subjects. A significant decrease of *Bifidobacterium*, *Clostridium lituseburense*, and *Faecalibacterium prausnitzii* and an increase in *Enterobacteriaceae* and *Escherichia coli* counts were found. Therefore, the supplementation with a probiotic to restore the imbalance in the gut microbiota might be a reasonable therapeutic option by downregulating the proinflammatory immune response in CD patients [90]. The design of specific probiotics comprises advanced genomic and metabolomics techniques using the interactions between the human body-microbiota and intra-microbiota, eventually leading to tailored specific probiotic therapies for microbiome regulation and health sustainability. 

Probiotics play an important role in preventing the overgrowth of potentially pathogenic bacteria and maintaining the integrity of the gut mucosal barrier. The beneficial effects of probiotics have been previously studied in adult patients with IBS. Oral administration of a probiotic mixture of *Lactobacillus plantarum* 14D-CECT 4528, *Lactobacillus casei, Bifidobacterium breve* Bbr8 LMG P-17501, *B. breve* Bl10 LMG P-17500, and *Bifidobacterium animalis* under randomized, double-blind, and placebo-controlled conditions showed the improvement in symptoms of adult CD patients with IBS [100]. In the future, microorganisms or even genetically engineered microorganisms could be used to act as living enzyme machinery as well as vectors for the delivery of endopeptidases capable of digesting gluten in the stomach, thereby allowing celiac patients to have a controlled dietary gluten intake [91,151].

In conclusion, probiotics are not expected to provide a rapid cure for complex diseases such as CD, but rather to alleviate the severity of symptoms [99]. More studies are needed to address how the gut microbiome can modulate or alter the course of the disease. To date, there are no guidelines available that recommend probiotic use in patients with CD. However, the data suggest a strong adjunctive role in the management of symptoms and bacterial overgrowth.

## 4. Clinical Trials

**Figure 3:**
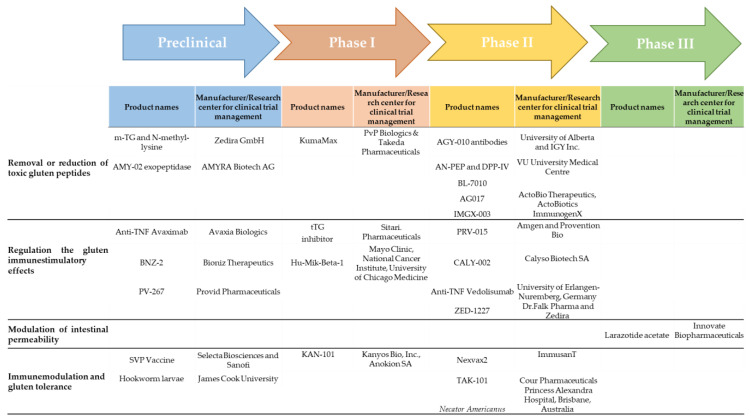
Clinical and preclinical development pipeline for CD. CD, celiac disease; PEP, prolyl endopeptidases; TNF, tumor necrosis factor; tTG, tissue transglutaminase.

Clinical endpoints are variables to quantify the potential effect of the treatment or intervention under study and reflect or characterize how a subject “feels, functions, or survives” [152]. Many major disease areas have established clinical trial endpoints because a fair number of registration trials have already been conducted and drugs approved for marketing. As in CD, there are no approved products and little experience, and agreed endpoints are lacking. Certain treatments in CD could control symptoms and prevent worsening of damage, while others are, at least initially, focused primarily on healing and maintenance of healing, with little effect on symptoms. Therefore, different endpoints or endpoint instruments are needed [153]. To date, only larazotide acetate is currently in phase III studies; most of them at phase II and a few phase I trials have explored its efficacy. Some therapies are being evaluated in preclinical trials and are postulated to be promising treatments for CD pathogenesis (Figure 3). We are facing many promising and emerging options for the treatment of CD. 

## 5. Conclusions

Although a GFD has been shown to be safe and effective in most celiac patients, the limitations caused by dietary gluten restriction and high gluten exposure rates raise the need to develop new therapies for CD. Different non-dietary therapeutic strategies are currently in the development phase and in clinical research, which could be a useful option in the medium- or long-term in patients with CD. To date, larazotide acetate is the most advanced experimental drug that has shown a reduction in symptoms as well as anti-tTG antibody titers. Promising PRV-015 immunotherapy requires more assays to establish rational targets for disease prevention. The use of glutenases as food preprocessors has proven to be very effective; however, the use of oral glutenases is perhaps the most accepted strategy for patients with CD and one of the most numerous options in terms of ongoing studies. All efforts are now being made to assess the effectiveness of these enzymes as a supplement to a GFD, highlighting the phase II results of IMGX-003 being very promising. Vaccine therapy has limitations, such as that it can engage only known or previously investigated immunogenic epitopes and effectiveness with the specific HLA-DQ2 genotype. However, if successful, it has the potential to have prolonged benefits on patients.

In addition, other many interesting drugs are in early research stages, such as tTG inhibitors, HLA blockers, and probiotics, although probiotics will probably need to be combined with long-term dietary changes. While several trials are ongoing or underway for CD, there is no consensus on outcome measures in CD patient trials.

Preventing the onset of CD entirely would be the most beneficial and desirable approach; however, recent approaches argue whether ingesting certain amounts of gluten plays a complementary or “adjuvant” role to a GFD and not as a substitute to a GFD in patients with CD. Nevertheless, some of these therapies could also be effective in other gluten-related pathologies in which a minimal amount of gluten is tolerable. 

Great efforts are ongoing to determine the effectiveness and the dose limit of gluten ingested in these therapies. It is also obvious that the possibility of using synergistic strategies could increase the maximum safe doses allowed for CD; therefore, this issue will be the next challenge.

## Figures and Tables

**Table 1:** Summary of strategies for CD grouped according to their goals.

Strategy	Goal	Therapy		References
Removal of toxic gluten peptides before reaching the intestine	Genetic modification of gluten-containing cereals	Genetically modified wheat flours		[59,60,61]
	Microbial gluten modification	Pretreatment with probiotic bacteria of the genus *Lactobacillus* (VSL#3)		[62]
		Pretreatment with microbial transglutaminase (m-TG) and N-methyl-lysine		[63]
	Masking of antigenic gluten capacity	Polymeric resins HEMA-co-SS		[64,65]
		AGY-010		[66]
	Luminal gluten detoxification	Prolyl endopeptidases (PEPs)	*Flavobacterium meningosepticum* (FM-PEP)	[67,68]
			*Myxococcus xanthus* (MX-PEP)	[69]
			*Sphingomonas capsulata* (SC-PEP)	[70,71]
			*Aspergillus niger* (AN-PEP)	[72]
		Gluten hydrolytic enzyme cocktail	SC-PEP and EPB-2 (ALV003)	[73]
			FM-PEP and EPB-2	[74]
		Subtilisin derived from *Rothia mucilaginosa* (Sub-A)		[75]
		Cysteine endopeptidase derived from *Hordeum vulgare* (EP-B2)		[21]
		Elastase derived from *Homo sapiens* (CEL-3B)		[22]
Regulation of the immunostimulatory effects of toxic gluten peptides	Immune response regulation	Inhibition of transglutaminase (ZED 1227)		[76]
		Blocker of HLA DQ binding to T-cells		[77]
		NK lymphocyte activation blocker: NKG2D receptor antagonists		[78]
		Lymphocyte recruitment blocker	Anti-α4 integrin (natalizumab)	[79]
			Anti-integrin α4β7 (vedolizumab)	
			Binding inhibitors CD40-CD40L	
			Binding inhibitors CXCL10- CXCR3	
			Binding inhibitors CCL25-CCR9	
		Anti-cytokines	Anti-IL-15, PRV-015, CALY-002 (AMG714)	[76,80]
			Anti-TNF-α (infliximab and adalimumab)	
			Anti-TNF- γ (fontolizumab)	
	Inhibition of the proinflammatory cascade	Anti-inflammatories (generic corticosteroids, budesonide, mesalazine)		[81]
Modulation of intestinal permeability	Barrier enhancing therapies	Larazotide acetate (AT-1001 and INN-202)		[82,83]
Immune modulation and induction of tolerance to gluten	Immunomodulation and gluten tolerance	Vaccine Nexvax2		[84,85]
		TAK-101 (CNP-101 and TIMP-GLIA)		[86]
		KAN-101		[87]
		Hookworm infection (*Necator americanus*)		[88]
		Mucosal tolerance due to genetic modification		[89]
Restoration of the imbalance in the gut microbiota	Probiotic supplementation	Microbial therapies		[90,91]

TNF, tumor necrosis factor; IgA, immunoglobulin A; Il-15, interleukin 15; NK, natural killer; PEP, prolyl endopeptidase; P-HEMA-co-SS, poly-hydroxyethylmethacrylateco-styrene sulfonate.

**Table 2:** Summary of glutenases used in enzyme therapy and classified according to origin of isolation, producer organism, and catalytic mechanism. ND, not determined.

Source of Enzymes	Peptidase Type	Organism	Isolated Enzyme	References
Bacterial peptidases	Prolyl endopeptidase	*S. capsulata*	SC-PEP	[68]
		*M. xanthus*	MX-PEP	[65]
		*F.meningosepticum*	FM-PEP	[66]
		*Chryseobacterium taeanense*	PEP 2RA3	[109]
	Subtilisin	*Rothia aeria*	ND	[112]
		*R. mucilaginosa*	Sub-A	[112]
		*Bacillus licheniformis*	ND	[113]
	Pseudolysin	*Pseudomonas aeruginosa*	lasB	[114]
	Thermolysin	*Bacillus thermoproteolyticus*	ND	[113]
	Serine peptidase	*Bacillus tequilensis*	ND	[115]
	ND	*Bacillus spp* GS 188	ND	[116]
	Serine carboxyl peptidase	*Actinoallomurus* A8	E40	[117]
Fungal peptidases	Prolyl endopeptidase	*A. niger*	AN-PEP	[72]
	Aspergillopepsin	*A. niger*	ASP	[118]
	Exopeptidase	*Aspergillus oryzae*	AO-DPP-IV	[119]
Plant peptidases	Cysteine endopeptidase	*H. vulgare*	EP-B2	[120]
		*Carica papaya*	Caricain	[121]
		*Triticum aestivum*	Triticain-α	[122]
		*H. vulgare*	HvPap-6 CysProt	[123]
Insect peptidases	Prolyl peptidase	*Rhizopertha dominica*	ND	[123]
	Prolidase	*Tenebrio molitor*	ND	[124]
Human peptidases	Elastase	*Homo sapiens*	CEL3B	[22]
		*Homo sapiens*	CEL2A	[22]
	Carboxypeptidase	*Homo sapiens*	CBPA1	[22]
